# Bovine leukemia virus reduces anti‐viral cytokine activities and NK cytotoxicity by inducing TGF‐β secretion from regulatory T cells

**DOI:** 10.1002/iid3.93

**Published:** 2016-01-18

**Authors:** Kosuke Ohira, Ayako Nakahara, Satoru Konnai, Tomohiro Okagawa, Asami Nishimori, Naoya Maekawa, Ryoyo Ikebuchi, Junko Kohara, Shiro Murata, Kazuhiko Ohashi

**Affiliations:** ^1^ Department of Disease ControlGraduate School of Veterinary MedicineHokkaido UniversitySapporo 060‐0818Japan; ^2^ Hokkaido Research OrganizationAgriculture Research Department, Animal Research CenterShintoku 081‐0038Japan

**Keywords:** BLV, IFN‐γ, NK cytotoxicity, perforin, TGF‐β, TNF‐α, Treg

## Abstract

CD4^+^CD25^high^Foxp3^+^ T cells suppress excess immune responses that lead to autoimmune and/or inflammatory diseases, and maintain host immune homeostasis. However, CD4^+^CD25^high^Foxp3^+^ T cells reportedly contribute to disease progression by over suppressing immune responses in some chronic infections. In this study, kinetic and functional analyses of CD4^+^CD25^high^Foxp3^+^ T cells were performed in cattle with bovine leukemia virus (BLV) infections, which have reported immunosuppressive characteristics. In initial experiments, production of the Th1 cytokines IFN‐γ and TNF‐α was reduced in BLV‐infected cattle compared with uninfected cattle, and numbers of IFN‐γ or TNF‐α producing CD4^+^ T cells decreased with disease progression. In contrast, IFN‐γ production by NK cells was inversely correlated with BLV proviral loads in infected cattle. Additionally, during persistent lymphocytosis disease stages, NK cytotoxicity was depressed as indicated by low expression of the cytolytic protein perforin. Concomitantly, total CD4^+^CD25^high^Foxp3^+^ T cell numbers and percentages of TGF‐β^+^ cells were increased, suggesting that TGF‐β plays a role in the functional declines of CD4^+^ T cells and NK cells. In further experiments, recombinant bovine TGF‐β suppressed IFN‐γ and TNF‐α production by CD4^+^ T cells and NK cytotoxicity in cultured cells. These data suggest that TGF‐β from CD4^+^CD25^high^Foxp3^+^ T cells is immunosuppressive and contributes to disease progression and the development of opportunistic infections during BLV infection.

## Introduction

The regulatory T cell (Tregs) types CD4^+^CD25^+^Foxp3^+^, Tr1, and γδ are important immune cell populations that limit immune responses and maintain host immune homeostasis 1. Among these, CD4^+^CD25^+^Foxp3^+^ T cells express the immunoglobulin cytotoxic T‐lymphocyte antigen 4 (CTLA‐4), the interleukin (IL)‐2 receptor CD25, and produce inhibitory cytokines such as IL‐10 and transforming growth factor (TGF)‐β 2, 3. CD4^+^CD25^+^Foxp3^+^ T cells express CD25 at high levels and sequester IL‐2 accordingly, rendering these T cells prone to apoptosis 4. IL‐10 induces the expression of programmed death ligand‐1 (PD‐L1), which acts as a ligand for the immune‐inhibitory receptor PD‐1 in dendritic cells, disturbs activation signals from CD28, and inhibits T cell activation 5. Similarly, TGF‐β inhibits the functions of T cells and antigen presenting cells in part by inhibiting the essential Th1 and Th2 development transcription factors T‐bet and GATA3 6. TGF‐β enhances the development of CD4^+^CD25^+^Foxp3^+^ T cells by inducing Foxp3 and inhibits natural killer (NK) cell cytotoxicity following activation by IL‐2, IL‐15, IL‐21, and type I IFN 7, 8, 9, 10. Although CD4^+^CD25^+^Foxp3^+^ T cells are important for host immune homeostasis, they reportedly facilitate progression of some chronic diseases by suppressing immune responses. Accordingly, CD4^+^CD25^+^Foxp3^+^ T cells from human immunodeficiency virus (HIV)‐infected patients clearly inhibited effector functions including IFN‐γ production and cytotoxicity, suggesting that CD4^+^CD25^+^Foxp3^+^ T cells compromise antiviral immune responses and favor viral replication in HIV infected individuals 11. Similarly, increased CD4^+^CD25^+^Foxp3^+^ T cell numbers were correlated with viral propagation during simian immunodeficiency virus (SIV)‐infection 12, 13. Previously, TGF‐β from CD4^+^CD25^+^Foxp3^+^ T cells represented a mechanism of infectious tolerance 14, was produced in abundance by CD4^+^CD25^+^Foxp3^+^ T cells, and was positively correlated with hepatitis virus levels in infected patients 15, 16, 17. Thus, CD4^+^CD25^+^Foxp3^+^ T cell‐mediated immune suppression may facilitate persistence of viral infections. Although CD4^+^CD25^+^Foxp3^+^ T cells have been identified as a regulatory T cell population in animals, few studies have assessed the functions of this cell population and the ensuing effects on chronic infections.

Bovine leukemia virus (BLV) is related to the human T‐cell leukemia retrovirus type 1 (HTLV‐1) and causes enzootic bovine leucosis (EBL) 18, 19. BLV infections are latent in the aleukemic (AL) state, but can emerge as persistent lymphocystosis (PL) with non‐malignant polyclonal expansion of CD5^+^ B‐cells that predominantly harbor BLV provirus, and rarely as malignant B‐cell lymphoma in various lymph nodes after long periods of latency 18. During BLV infection, reduced cellular immune responses play major roles in disease progression and lead to increased susceptibility to other infections 20, 21, 22. In previous studies, several factors were shown to play critical roles in the suppression of cellular immune responses and disease progression in BLV‐infections 23, 24. Subsequently, we reported that proportions of Foxp3^+^CD4^+^ cells were positively correlated with numbers of lymphocytes, virus titers, and virus loads in BLV infected cattle but were inversely correlated with IFN‐γ expression 25, 26. However, although Foxp3^+^CD4^+^ cells downregulate antiviral cytokines, their influence on BLV propagation remains uncharacterized. Thus, to elucidate the roles of CD4^+^CD25^high^Foxp3^+^ T cells in BLV‐mediated immunosuppression and disease progression, we performed kinetic and functional analysis of TGF‐β from CD4^+^CD25^high^Foxp3^+^ T cells in BLV infected cattle.

## Material and Methods

### Bovine samples

Bovine peripheral blood samples were obtained from Holstein breed cattle and were maintained at the Field Science Center for Northern Biosphere, Hokkaido University, or at dairy farms in Hokkaido. BLV‐infected cattle were diagnosed at the Veterinary Teaching Hospital, Graduate School of Veterinary Medicine, Hokkaido University, between 2013 and 2014. BLV infections were identified using nested‐PCR, and provirus loads were confirmed using quantitative real‐time PCR as described previously 24. Lymphocyte numbers in BLV‐positive cattle were counted using Celltac α MEK‐6450 (NIHON KOHDEN, Tokyo, Japan) and animals were diagnosed with PL when two consecutive lymphocyte counts were more than 7,500/µL 27.

### Cytokine assay

Bovine whole blood was cultivated in the presence of mouse anti‐bovine CD3 antibody (2 µg/mL; MM1A: VMRD, Pullman, WA), mouse anti‐bovine CD28 antibody (2 µg/mL; AbD Serotec, Oxford, UK), and recombinant bovine IL‐2 (10 ng/mL; Kingfisher Biotech, Saint Paul, MN) in 12‐well plates. After 24 h, supernatants were harvested and IFN‐γ (Mabtech, Nacka Strand, Sweden) and TNF‐α (Kingfisher Biotech) production was determined using ELISA kits with a microplate reader MTP‐650FA (Corona Electric, Ibaraki, Japan) at an absorbance of 450 nm.

Bovine peripheral blood mononuclear cells (PBMCs) were purified using density gradient centrifugation on Percoll solution (GE Healthcare UK, Buckinghamshire, UK). The purified PBMCs were cultured in RPMI 1640 medium (Sigma‐Aldrich, St Louis, MO) containing 10% FCS and 1% L‐glutamine (Invitrogen, Carlsbad, CA) at 37°C under the conditions described above. Brefeldin A (10 µg/mL; Sigma‐Aldrich) was added to the culture medium 6 h before harvest, and harvested cells were washed with 10%‐inactivated caprine serum in phosphate buffered saline (PBS) and were stained with FITC‐conjugated mouse anti‐bovine CD4 antibody (AbD serotec) and mouse anti‐bovine CD8 antibody (AbD serotec), which were labeled with Alexa Fluor 647 using Zenon mouse IgG Labeling Kits (Life Technologies, Carlsbad, CA). After staining for 30 min at 4°C, cells were treated with FOXP3 Fix/Perm Buffer (BioLegend, San Diego, CA) and were stained with PE‐conjugated mouse anti‐bovine IFN‐γ antibody (AbD serotec), biotin‐conjugated mouse anti‐bovine TNF‐α antibody (AbD serotec), and APC‐eFluor780‐conjugated Streptavidin as secondary antibody for TNF‐α for 20 min at 4°C. Stained cells were analyzed using FACS Verse (BD Biosciences, San Jose, CA) and FCS Express 4 (De Novo Software, CA).

### Flow cytometric analyses of Tregs

Bovine blood was treated with red blood cell lysis buffer containing 8.26 mg/mL NH_4_Cl, 1.19 mg/mL NaHCO_3_, and 37.8 μg/mL 2Na‐EDTA (pH 7.3), and then washed with PBS. CD4^+^CD25^high^Foxp3^+^ T cells were then stained with FITC‐conjugated mouse anti‐bovine CD4 antibody (AbD serotec), Alexa Fluor 647 (Life Technologies) labeled mouse anti‐bovine CD25 antibody (AbD serotec), and mouse anti‐bovine CD3 antibody (VMRD), which was labeled with PE using a Zenon mouse IgG Labeling Kit (Life Technologies). After staining, cells were treated with FOXP3 Fix/Perm Buffer (BioLegend) and were stained with PerCP‐Cy5.5‐conjugated rat anti‐bovine Foxp3 antibody (eBioscience, San Diego, CA, USA). PerCP‐Cy5.5‐conjugated rat IgG2a κ chain isotype control (eBioscience) was used as negative control.

WC1^+^TCRδ^+^ T cells identified by staining with PE‐labeled mouse anti‐bovine CD3 antibody (VMRD), Alexa Fluor 647‐labeled mouse anti‐bovine TCRδ antibody (VMRD), mouse anti‐bovine WC1 antibody (AbD serotec) and its corresponding secondary antibody, and FITC‐conjugated rat anti‐murine IgG2a antibody (BD Biosciences).

### Detection of TGF‐β and IL‐10 in CD4^+^CD25^high^Foxp3^+^ T cells and WC1^+^TCR^+^ T cells

PBMCs were cultured for 6 h in the presence of Concanavalin A (5 µg/mL; Sigma‐Aldrich) and Brefeldin A (10 µg/mL; Sigma‐Aldrich) and were harvested and stained as above to detect CD4^+^CD25^high^Foxp3^+^ T cells and WC1^+^TCR^+^ T cells. To detect TGF‐β and IL‐10, cells were stained with PE‐conjugated mouse anti‐human TGF‐β antibody (R&D Systems, Minneapolis, MI), Biotin‐conjugated mouse anti‐bovine IL‐10 antibody (AbD serotec), and secondary APC‐eFluor 780‐cojugated streptavidin antibody (eBioscience). Stained cells were analyzed using FACS Verse and FCS Express 4 as described above.

### T‐cell bioassay with bovine recombinant TGF‐β

PBMCs were pre‐cultured for 2 h in the presence of recombinant bovine TGF‐β (1 ng/mL; Genorise Scientific, Glen Mills, PA, USA) and were then incubated with mouse anti‐bovine CD3 antibody (2 µg/mL; VMRD), mouse anti‐bovine CD28 antibody (2 µg/mL; AbD serotec), and recombinant bovine IL‐2 (10 ng/mL; Kingfisher Biotech) alone or with BLV‐gp51 peptide mix (5 µg/mL) 28 for 24 h. Brefeldin A (10 µg/mL; Sigma‐Aldrich) was added to the medium 6 h before cell harvest, and IFN‐γ and TNF‐α were determined in CD4^+^ cells using flow cytometry as described above.

### Detection of NK cells using flow cytometry

To detect NK cells in peripheral blood, purified PBMCs were stained with mouse anti‐bovine NKp46 antibody (AbD serotec), mouse anti‐bovine CD3 antibody (Accurate, Westbury, NY), and mouse anti‐bovine IgM antibody (AbD serotec). CD3^‐^IgM^‐^NKp46^+^ NK cell numbers were then determined accordingly and were additionally stained with FITC‐conjugated mouse anti‐bovine CD69 antibody (AbD serotec) to determine their activation states. The presence of anti‐viral factors was determined after treating cells with FOXP3 Fix/Perm Buffer (BioLegend) followed by staining with PerCP‐Cy5.5‐conjugated mouse anti‐human perforin antibody (BioLegend). PerCP‐Cy5.5‐conjugated mouse IgG2a κ chain isotype (BioLegend) was used as a negative control.

To determine IFN‐γ production by NK cells, PBMCs were cultivated in the presence of recombinant bovine IL‐2 (10 ng/mL; Kingfisher Biotech) and recombinant human IL‐12 (400 pg/mL; eBioscience) for 72 h. Brefeldin A (10 µg/mL; Sigma‐Aldrich) was then added to the medium 6 h before cell harvest. Harvested cells were treated with FOXP3 Fix/Perm Buffer (BioLegend) as described above and were then stained with PE‐conjugated mouse anti‐bovine IFN‐γ antibody (AbD serotec). The PE‐conjugated mouse IgG1 isotype (AbD serotec) was used as a negative control, and stained cells were analyzed using flow cytometry as described above.

### NK cytotoxicity assay with bovine TGF‐β

Effector CD3^‐^IgM^‐^NKp46^+^ cells were purified from PBMCs using a MoFlo^TM^ cell sorter (Beckman Coulter, Brea, CA) with the antibodies described above, and cell purity was confirmed using FACS Verse (BD Biosciences). Purified NK cells (more than 98% purity) were cultivated in the presence of recombinant bovine IL‐2 (10 ng/mL; Kingfisher Biotech) for 16 h. Target P815 cells 29 were pretreated with 0.1% bovine serum albumin (BSA) in PBS containing 0.2 µM carboxyfluorescein diacetate succinimidyl ester (CFSE)(Life technologies) for 15 min at room temperature. After washing with RPMI 1640 containing fetal calf serum (FCS), P815 cells were incubated with 0.1% BSA–PBS containing mouse anti‐bovine NKp46 antibody (AbD serotec) or mouse IgG1 isotype control (Beckman Coulter) for 20 min. NK and P815 cells were then co‐cultured for 5 h at ratios of NK (E) to P815 (T) cells of 0:1, 2:1, and 4:1. Harvested cells were washed with 0.1% BSA–PBS and were stained with 7‐amino‐actinomycin D (7‐AAD, BD Bioscience) for 10 min and analyzed using FACS Verse (BD Bioscience). Cytotoxicity (%) was calculated as follows: (% dead cells − % dead cells at E:T = 0:1) / (100 − % of dead cells at E:T = 0:1) × 100. To evaluate cytotoxic effects of TGF‐β, CD3^‐^IgM^‐^NKp46^+^ cells were cultured in the presence of recombinant bovine IL‐2 (10 ng/mL; Kingfisher Biotech) alone or with recombinant bovine TGF‐β (5 ng/mL; Genorise Scientific) for 40 h. Cytotoxicity (%) was then determined as above at a NK:P815 cell ratio of 2:1.

### Statistics

Two‐tailed paired comparisons were performed using the Wilcoxon signed rank test or Spearman rank correlation coefficients. Other paired comparisons were performed using the U test, and comparisons of the three‐armed trial were made using the Steel test. Two‐tailed unpaired comparisons were performed using the Student's *t*‐test and Welch's *t*‐test, and correlations were identified using the Spearman rank test.

## Results

### Reduced anti‐viral cytokine production during BLV‐infection

**Figure 1:**
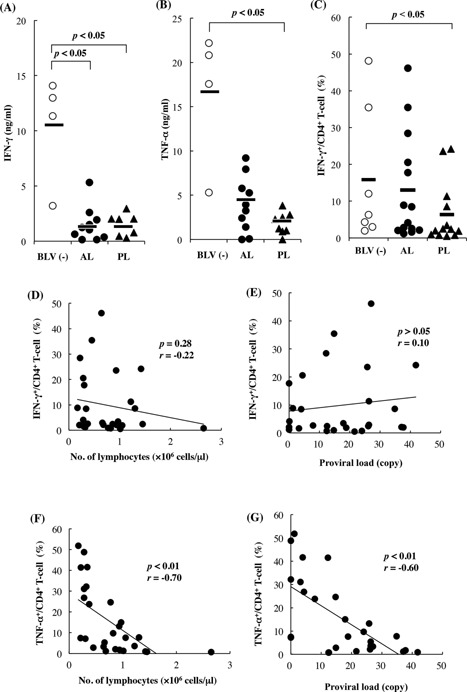
Reduced anti‐viral cytokine production in BLV‐infected cattle. IFN‐γ (A) and TNF‐α (B) in the supernatants of cultured PBMCs from BLV‐infected aleukemic (AL: *n* = 9) or persistent lymphocytotic (PL: *n* = 7) cattle and uninfected cattle (*n* = 4) were determined using ELISA. Detection of IFN‐γ producing CD4^+^ T cells in BLV‐uninfected and BLV‐infected cattle at different disease stages (C: BLV(−): *n* = 7, AL: *n* = 14, PL: *n* = 13). Correlations between IFN‐γ producing CD4^+^ T cells and lymphocyte numbers (D) and proviral loads (E), TNF‐α producing CD4^+^ T cells, lymphocyte numbers (F), and proviral loads (G) in BLV‐infected cattle (*n* = 27). Lymphocyte numbers in BLV‐infected cattle were counted using Celltac α MEK‐6450 and animals were diagnosed with PL when at least two consecutive lymphocyte counts were >7,500/µL; proviral loads were quantified using real‐time PCR. IFN‐γ‐ and TNF‐α‐producing CD4^+^ T cells in BLV‐infected cattle were detected using flow cytometry.

To verify the effects of BLV on anti‐viral cytokine production, we used ELISA and flow cytometry to measure IFN‐γ and TNF‐α in PBMCs isolated from naturally infected cattle with aleukemic BLV or persistent lymphocytotic BLV and uninfected cattle. Mean IFN‐γ production in AL and PL cattle was significantly lower than in uninfected cattle (*p* < 0.05; Fig. 1A). Similarly, TNF‐α production was significantly decreased in AL and PL cattle compared with uninfected cattle (Fig. 1B). To confirm these reductions in cytokine production, proportions of CD4^+^ PBMCs secreting IFN‐γ and TNF‐α were determined using flow cytometry. Although proportions of IFN‐γ secreting CD4^+^ cells in PL cattle were significantly (*p* < 0.05) lower than those in uninfected cattle (Fig. 1C), no correlations between the IFN‐γ secreting CD4^+^ cells and the numbers of lymphocyte (Fig. 1D) or proviral loads (Fig. 1E) were found in infected cattle. In contrast, proportions of TNF‐α secreting CD4^+^ cells were negatively correlated with numbers of lymphocytes (Fig. 1F; *p* < 0.01) and proviral loads (Fig. 1G; *p* < 0.01).

### Subpopulations of CD4^+^CD25^high^Foxp3^+^ T cells and WC1^+^TCRγ^+^ T cells

**Figure 2:**
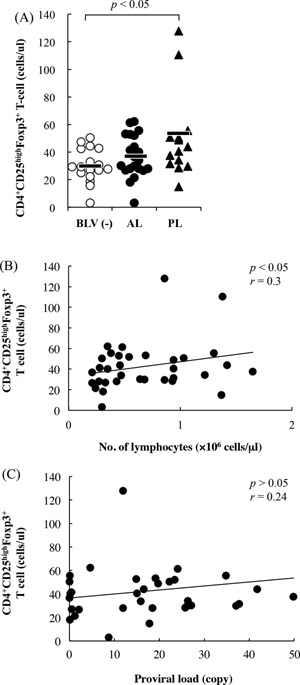
(A) The number of CD4^+^CD25^high^Foxp3^+^ T cells in cattle (BLV(−): *n* = 17, AL: *n* = 21, PL: *n* = 13). Detection of CD4^+^CD25^high^Foxp3^+^ T cells in the fresh PBMCs derived from BLV‐uninfected and BLV‐infected cattle at different disease stages using flow cytometry analysis. Correlation between CD4^+^CD25^high^Foxp3^+^ T cells and lymphocyte numbers (B) and proviral loads (C) in BLV‐infected cattle (*n* = 34).

To investigate Treg subpopulation during BLV‐infection, CD4^+^CD25^high^Foxp3^+^ T cells were detected among PBMCs isolated from BLV‐infected cattle using flow cytometry. In these experiments, numbers of CD4^+^CD25^high^Foxp3^+^ T cells did not differ significantly between AL cattle and uninfected cattle (Fig. 2A). However, numbers of CD4^+^CD25^high^Foxp3^+^ T cells were significantly higher in PL cattle than in uninfected cattle (Fig. 2A) and were positively correlated with numbers of lymphocytes in infected cattle (Fig. 2B).

**Figure 3:**
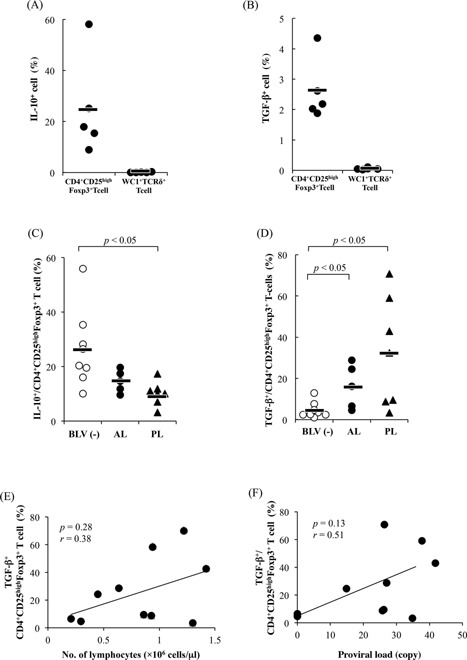
Increasing TGF‐β production from CD4^+^CD25^high^Foxp3^+^ T cells in BLV‐infected cattle. IL‐10 (A) and TGF‐β (≡) were detected in CD4^+^CD25^high^Foxp3^+^ T cells and WC1^+^TCRδ^+^ T cells. PBMCs from normal cattle (*n*=5) were cultivated for 6 h in the presence of concanavalin A, and IL‐10‐ and TGF‐β‐producing cells were detected using flow cytometry. IL‐10‐ (C) and TGF‐β‐producing (D) cells were detected among CD4^+^CD25^high^Foxp3^+^ T cells in BLV‐uninfected (*n* = 8) and BLV‐infected cattle at different disease stages (AL: *n* = 5, PL: *n* = 7) using flow cytometry. Correlation between TGF‐β−producing CD4^+^CD25^high^Foxp3^+^ T cells and lymphocyte numbers (E) and proviral loads (F) in BLV‐infected cattle (*n* = 10).

Moreover, numbers of CD4^+^CD25^high^Foxp3^+^ T cells were positively correlated with proviral loads, although no significant differences were found among the infected cattle (Fig. 2C). In a previous study, bovine WC1^+^ T cells but not CD4^+^CD25^+^ Foxp3^+^ T cells were shown to act as immune regulatory cells ex vivo 30. Thus, to investigate the kinetics of WC1^+^ T cells during BLV‐infection, the numbers of WC1^+^TCRγ^+^ cells were determined among PBMCs from BLV‐infected cattle using flow cytometry. However, WC1^+^TCRγ^+^ cell numbers did not differ significantly between infected cattle and uninfected cattle (data not shown). In addition, whereas WC1^+^TCRγ^+^ cells did not produce the immunoinhibitory cytokines IL‐10 and TGF‐β, CD4^+^CD25^+^ Foxp3^+^ T cells did produce these cytokines (Fig. 3A and B). Further experiments showed that TGF‐β‐secreting CD4^+^CD25^+^ Foxp3^+^ T cell proportions were significantly higher in AL and PL cattle than in uninfected cattle (*p* < 0.05, Fig. 3D), whereas IL‐10 production was lower (Fig. 3C). As shown in Fig 3E and F, TGF‐β secreting CD4^+^CD25^+^ Foxp3^+^ T cell numbers tend to increase with increasing numbers of lymphocytes and proviral loads in BLV‐infected cattle, although significant differences were not observed.

### TGF‐β mediated reductions in anti‐viral cytokines

**Figure 4:**
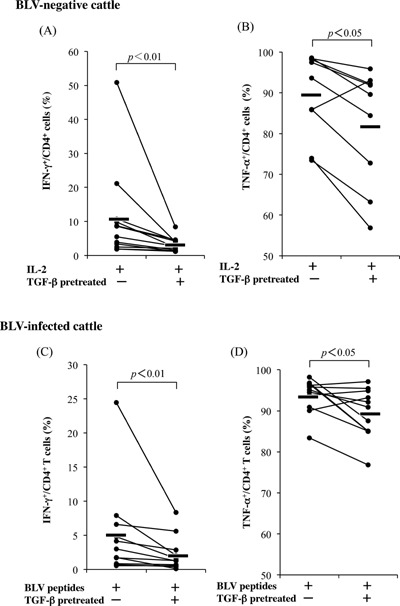
Inhibition of IFN‐γ and TNF‐α production from CD4^+^ T cells by TGF‐β. PBMCs from normal cattle (IFN‐γ: *n* = 12, TNF‐α: *n* = 9) and BLV‐infected cattle (*n* = 11) were pretreated with TGF‐β for 2 h and were cultivated with IL‐2 alone (A and B) or synthesized peptides from the BLV envelop region (C and D). IFN‐γ‐ or TNF‐α‐producing CD4^+^ T cells were detected using flow cytometry.

As shown Figures 1 and 3, TGF‐β secreting CD4^+^CD25^+^ Foxp3^+^ T cells were increased in BLV‐infected cattle with reductions of IFN‐γ and TNF‐α production in CD4^+^ T cells. Thus, the effects of TGF‐β on cytokine production were investigated in CD4^+^ T cells. In these experiments, TGF‐β inhibited IFN‐γ and TNF‐α production by CD4^+^ T cells in infected and uninfected cattle (Fig. 4A and B), suggesting a direct influence of TGF‐β secreting CD4^+^CD25^+^ Foxp3^+^ T cells on cytokine production by anti‐BLV‐specific and non‐specific CD4^+^T cells.

### Reduced NK activity during BLV‐infection

**Figure 5:**
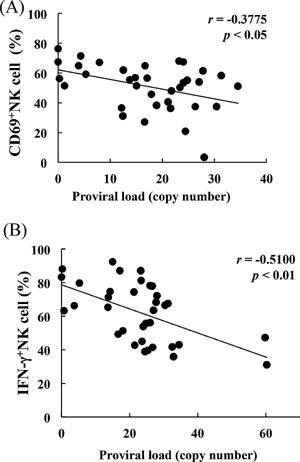
Negative correlations between CD69^+^ NK cells (A) and IFN‐γ production from NK cells (B) and proviral loads in BLV‐infected cattle (*n* = 35). CD69 expression and IFN‐γ production were detected using flow cytometry.

**Figure 6:**
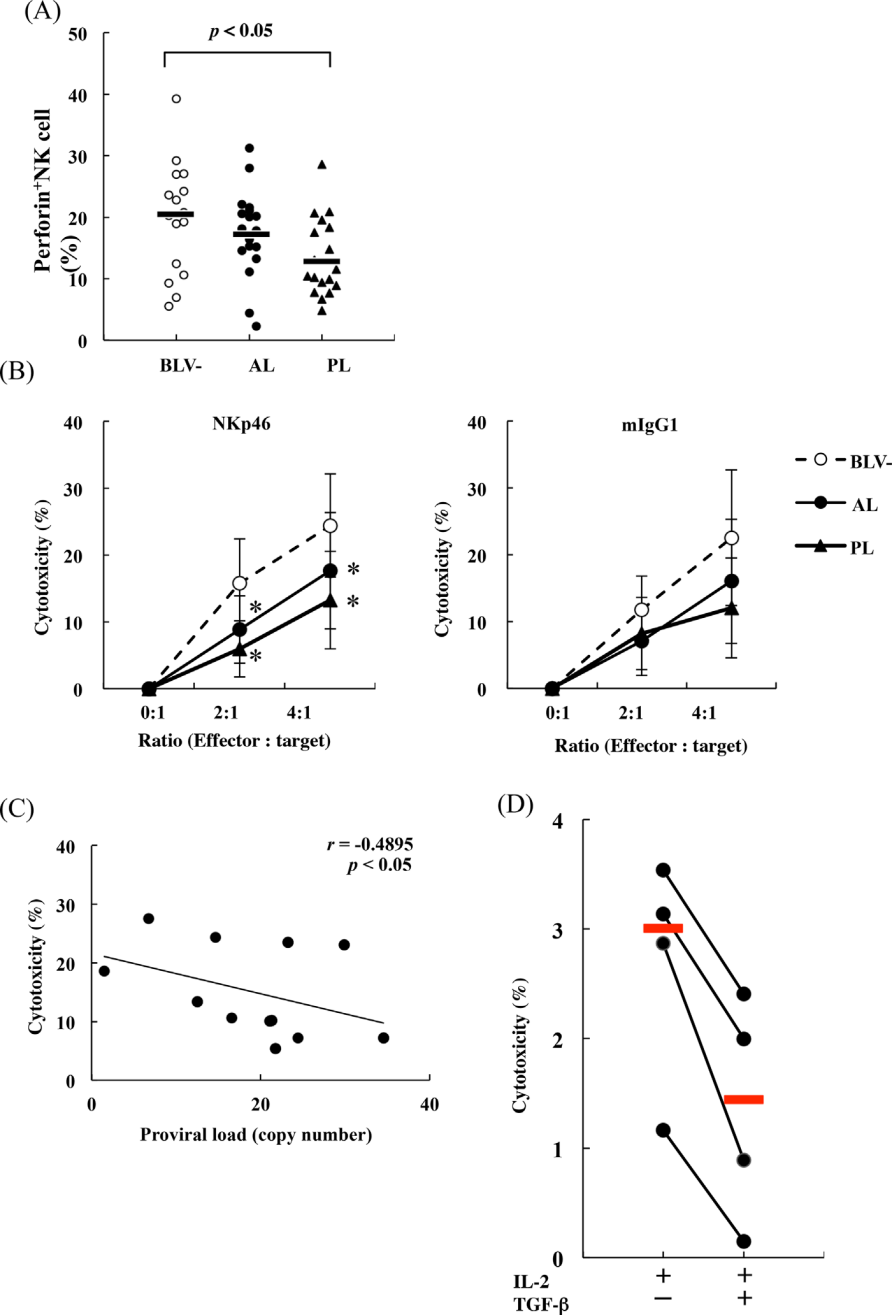
Decreased perforin production (A: BLV(−): *n* = 16, AL: *n* = 18, PL: *n* = 18) and cytotoxicity (B BLV(−): *n* = 7, AL: *n* = 5, PL: *n* = 7) of NK cells in BLV‐infected cattle with PL. Asterisks donate significant differences between the cytotoxicity of NK cells from infected and uninfected cattle (*p < 0.05); (C) Negative correlations between NK cytotoxicity and proviral loads in the BLV‐infected cattle (*n* = 12); (D) Inhibition of NK cytotoxicity by TGF‐β (*n* = 4).

In this study, kinetics and functions of NK cells, and the effects of TGF‐β on NK activity were investigated to determine their involvement in innate immunity of BLV‐infected cattle. Although NK cell numbers were not affected by BLV infection (data not shown), activated CD69^+^NK cells, and their capacity to produce IFN‐γ were inversely correlated with BLV proviral loads (Fig. 5A and B). Furthermore, NK cytotoxicity in PL cattle was depressed, reflecting low expression of the cytolytic protein perforin (Fig. 6A and B), and NK cytotoxicity was inversely correlated with BLV proviral loads in infected cattle (Fig. 6C). NK cytotoxicity was also inhibited by recombinant TGF‐β (Fig. 6D).

## Discussion

Dysfunctions of cell‐mediated immunity, such as limited anti‐viral cytokine expression and impaired cell proliferation, have been documented well in BLV‐infected cattle 20, 21, 22. Moreover, cell‐mediated immune dysfunction accelerates disease progression during BLV‐infection as indicated in previous studies showing that IFN‐γ plays important roles in protective mechanisms against BLV propagation in infected animals 23, 24, 28. In addition, the ensuing immune disorder may contribute to the development of opportunistic infections in BLV‐infected cattle 31, 32. Accordingly, BLV likely accelerates disease progression during *Mycobacterium avium* (subspecies *paratuberculosis*) mediated Johne's disease 33. We recently showed that proportions of Foxp3^+^CD4^+^ cells correlate positively with increased lymphocyte numbers, virus titers, and virus loads, and inversely correlate with IFN‐γ mRNA expression 25. Moreover, increased TGF‐β mRNA expression was correlated with Treg numbers 26, suggesting that bovine Foxp3^+^CD4^+^ T cells have immunosuppressive functions during BLV infection. In the present study, we investigated Treg functions by correlating CD4^+^CD25 ^high^Foxp3^+^ T cell numbers with T cell responses and NK activity in BLV‐infected cattle. Moreover, bioassays with recombinant bovine TGF‐β confirmed that inhibition of cell‐mediated immunity follows increased TGF‐β from increasing CD4^+^CD25^high^Foxp3^+^ T cell numbers in BLV‐infected cattle.

In further experiments, anti‐virus cytokine production was reduced as was shown in our previous reports 23, 24, 28. Moreover, CD4^+^CD25^high^Foxp3^+^ T cell numbers were increased in conjunction with increasing proportions of TGF‐β‐secreting CD4^+^CD25^high^Foxp3^+^ T cells, leading to correlations with increased proviral loads in BLV‐infected cattle, as shown previously 25, 26. Bovine WC1^+^ T cells rather than CD4^+^CD25^+^Foxp3^+^ T cells reportedly act as immune regulatory cells 30, 34, warranting investigations of WC1^+^ T cell kinetics during BLV‐infection. Among PBMCs from BLV‐infected cattle, WC1^+^TCRγ^+^ cells were present with CD4^+^CD25^+^Foxp3^+^ T cells but their numbers did not differ between BLV‐infected and BLV‐uninfected cattle (data not shown). In addition, WC1^+^TCRγ^+^ cells did not produce the immune‐inhibitory cytokines IL‐10 and TGF‐β, whereas CD4^+^CD25^+^Foxp3^+^ T cells did. Moreover, proportions of TGF‐β secreting CD4^+^CD25^high^Foxp3^+^ T cells in AL and PL cattle were significantly higher than in uninfected cattle, although IL‐10 production was lower. IL‐10 is recognized as a major immunoinhibitory cytokine that downregulates immune responses during chronic disease progression. Accordingly, increased IL‐10 expression has been correlated with disease progression during BLV infection 35, 36, 37, 38. The present data indicate that IL‐10 is produced by other cells, such as macrophages, but is not secreted by CD4^+^CD25^high^Foxp3^+^ T cells 35, 36. In contrast, although sample numbers were limited, increased TGF‐β secretion in CD4^+^CD25^high^Foxp3^+^ T cells was correlated with increased numbers of lymphocytes and proviral loads in BLV‐infected cattle, corroborating previously reported positive correlations between TGF‐β mRNA expression and Treg cell numbers 26. Hence, TGF‐β is likely involved in the observed deficits of anti‐viral cytokines and clearly inhibited cytokine production from isolated CD4^+^ T cells after stimulation with CD3 and CD28 antibodies. In addition, bovine TGF‐β inhibited anti‐viral cytokine production from BLV‐antigen‐specific CD4^+^ T cells, suggesting that TGF‐β is involved in immunosuppressive functions of virus‐specific and non‐virus‐specific T cells. However, IFN‐γ‐ or TNF‐α−secreting CD8^+^ T cell numbers were not correlated with disease progression in the present infected cattle (data not shown), warranting further investigation. Potentially, the cytolytic proteins perforin and granzyme are involved in anti‐viral functions of CD8^+^ T cells and could be included in future studies of T cell dysfunction in BLV‐infected cattle.

NK cells play important roles in immune responses and eliminate tumor and infected cells by releasing cytotoxic granules and pro‐inflammatory cytokines 39. Accordingly, NK cell dysfunction during HIV infection has been implicated in disease progression following observations of decreased NK cell activation in viremic patients 40. Previous studies have also shown that NK cytotoxicity is enhanced by IFN‐γ 10 and that TGF‐β strongly inhibits NK cytotoxicity 8, 9. In agreement, NK cytotoxicity was inversely correlated with TGF‐β in tumor patients 41. In the present study, TGF‐β secreting CD4^+^CD25^high^Foxp3^+^ T cell numbers were increased, and bovine TGF‐β inhibited the production of the NK cell stimulators IFN‐γ and TNF‐α. Moreover, although numbers of NK cells did not vary with BLV infection (data no shown), their capacity to produce IFN‐γ was inversely correlated with BLV proviral loads in infected cattle as immune dysfunction of NK cells in HIV 40. CD69 has also been positively correlated with IFN‐γ production and NK cytotoxicity 42, 43 and was inversely correlated with the BLV proviral loads in the present study. Hence, NK cytotoxicity may be reduced during BLV disease progression as indicated by the present observations of depressed NK cytotoxicity and low expression of perforin in PL cattle. Critically, NK cytotoxicity was clearly inhibited by TGF‐β and the expression of activating receptor NKp46 was downregulated in neoplastic lymph nodes, suggesting that inactivated NK cells enhance tumor formation during BLV‐infection by inducing TGF‐β secretion from CD4^+^CD25^+^ Foxp3^+^ T cells.

Among numerous characterized immune inhibitory responses in BLV‐infection, TGF‐β production from increasing numbers of Tregs is clearly significant. Although the mechanisms behind increasing CD4^+^CD25^high^Foxp3^+^ T cell numbers during BLV infection remains unknown, the HIV envelope protein gp120 reportedly inhibited apoptosis of Treg after binding CD40 44, and the nuclear HTLV‐I protein Tax induced aberrant expression of IL‐2, Bcl‐X_L_, and CD40 in virus infected T cells 45, 46. Hence, viral factor(s) may increase CD4^+^CD25^high^Foxp3^+^ T cell numbers in BLV‐infected cattle, although BLV infects B cells whereas HIV and HTLV‐I infect T cells. In a previous study, PD‐L1 was associated with stable expression of Foxp3 in CD4^+^CD25^+^Foxp3^+^ T cells, and enhanced Treg function 47. Moreover, PD‐L1 promoted the development of CD4^+^CD25^+^Foxp3^+^ T cells from naïve CD4^+^ T cells in the presence of TGF‐β 47. Previously, we reported that PD‐L1 expression in infected B cells increases with disease progression during BLV‐infection 24. However, although the effects of PD‐1 expression on CD4^+^CD25^high^Foxp3^+^ T cells remains unclear, these data suggest that the PD‐1/PD‐L1 pathway is involved in increased CD4^+^CD25^high^Foxp3^+^ T cell numbers, and warrant further studies to determine correlations between the PD‐1/PD‐L1 pathway and Treg function in infected cattle.

In conclusion, the present experiments show that TGF‐β secretion from CD4^+^CD25^high^Foxp3^+^ T cells is involved in immunosuppression and disease progression during BLV infection. Hence, CD4^+^CD25^high^Foxp3^+^ T cells may contribute susceptibility of BLV‐infected cattle to opportunistic infections, warranting further investigations of the immunosuppressive effects of CD4^+^CD25^high^Foxp3^+^ T cells and of related therapeutic strategies for opportunistic infections in BLV‐infected cattle.

## Conflicts of Interest

None declared.

## Author Contributions

KO and AN performed all of the experiments, analyzed the data, and drafted the manuscript. SK participated in the experimental design, analyzed data, and helped to draft the manuscript. TO, RI, AN, and NM participated in some experiments and sample collection. JK participated in sample collection. SM assisted with data analysis and provided overall guidance for the studies. KO supervised the study and reviewed the manuscript. All authors read and approved the final manuscript.

## References

[1] Jonuleit, H. , and E. Schmitt . 2003 The regulatory T cell family: distinct subsets and their interrelations. J. Immunol. 171:6323–6327. 14662827 10.4049/jimmunol.171.12.6323

[2] Oderup, C ., L. Cederbom , A. Makowska , C. M. Cilio , and F. Ivars . 2006 Cytotoxic T lymphocyte antigen‐4‐dependent down‐modulation of costimulatory molecules on dendritic cells in CD4^+^ CD25^+^ regulatory T‐cell‐mediated suppression. Immunology 118:240–249. 16771859 10.1111/j.1365-2567.2006.02362.x PMC1782280

[3] Joetham, A ., K. Takeda , K. Takada , C. Taube , N. Miyahara , S. Matsubara , T. Koya , Y. H. Rha , A. Dakhama , and E. W. Gelfand . 2007 Naturally occurring lung CD4^+^CD25^+^ T cell regulation of airway allergic responses depends on IL‐10 induction of TGF‐beta. J. Immunol. 178:1433–1442. 17237391 10.4049/jimmunol.178.3.1433

[4] Pandiyan, P ., L. Zheng , S. Ishihara , J. Reed , and M. J. Lenardo . 2007 CD4^+^CD25^+^Foxp3^+^ regulatory T cells induce cytokine deprivation‐mediated apoptosis of effector CD4^+^ T cells. Nat. Immunol. 8:1353–1362. 17982458 10.1038/ni1536

[5] Saraiva, M ., and A. O'Garra . 2010 The regulation of IL‐10 production by immune cells. Nat. Rev. Immunol. 10:170–181. 20154735 10.1038/nri2711

[6] Letterio, J. J. , and A. B. Roberts . 1998 Regulation of immune responses by TGF‐beta. Annu. Rev. Immunol. 16:137–161. 9597127 10.1146/annurev.immunol.16.1.137

[7] Chen, W. , W. Jin , N. Hardegen , K. J. Lei , L. Li , N. Marinos , G. McGrady , and S. M. Wahl . 2003 Conversion of peripheral CD4^+^CD25^‐^ naive T cells to CD4^+^CD25^+^ regulatory T cells by TGF‐beta induction of transcription factor *Foxp3* . J. Exp. Med. 198:1875–1886. 14676299 10.1084/jem.20030152 PMC2194145

[8] Bellone, G ., M. Aste‐Amezaga , G. Trinchieri , and U. Rodeck . 1995 Regulation of NK cell functions by TGF‐β1. J. Immunol. 155:1066–1073. 7636180

[9] Rook, A. H. , J. H. Kehrl , L. M. Wakefield , A. B. Roberts , M. B. Sporn , D. B. Burlington , H. C. Lane , and A. S. Fauci . 1986 Effects of transforming growth factor β on the functions of natural killer cells: depressed cytolytic activity and blunting of interferon responsiveness. J. Immunol. 136:3916–3920. 2871107

[10] Zwirner, N. W. , and C. I. Domaica . 2010 Cytokine regulation of natural killer cell effector functions. Biofactors 36:274–288. 20623510 10.1002/biof.107

[11] Kinter, A. L. , R. Horak , M. Sion , L. Riggin , J. McNally , Y. Lin , R. Jackson , A. O'shea , G. Roby , C. Kovacs , et al. 2007 CD25^+^ regulatory T cells isolated from HIV‐infected individuals suppress the cytolytic and nonlytic antiviral activity of HIV‐specific CD8^+^ T cells *in vitro* . AIDS Res. Hum. Retroviruses 23:438–450. 17411377 10.1089/aid.2006.0162

[12] Epple, H. J. , C. Loddenkemper , D. Kunkel , H. Tröger , J. Maul , V. Moos , E. Berg , R. Ullrich , J. D. Schulzke , H. Stein , et al.. 2006 Mucosal but not peripheral FOXP3^+^ regulatory T cells are highly increased in untreated HIV infection and normalize after suppressive HAART. Blood 108:3072–3078. 16728694 10.1182/blood-2006-04-016923

[13] Estes, J. D. , Q. Li , M. R. Reynolds , S. Wietgrefe , L. Duan , T. Schacker , L. J. Picker , D. I. Watkins , J. D. Lifson , C. Reilly , et al. 2006 Premature induction of an immunosuppressive regulatory T cell response during acute simian immunodeficiency virus infection. J. Infect. Dis. 193:703–712. 16453267 10.1086/500368

[14] Jonuleit, H ., E. Schmitt , H. Kakirman , M. Stassen , J. Knop , and A. H. Enk . 2002 Infectious tolerance: human CD25^+^ regulatory T cells convey suppressor activity to conventional CD4^+^ T helper cells. J. Exp. Med. 196:255–260. 12119350 10.1084/jem.20020394 PMC2193929

[15] Yang, G ., A. Liu , Q. Xie , T. B. Guo , B. Wan , B. Zhou , and J. Z. Zhang . 2007 Association of CD4^+^CD25^+^Foxp3^+^ regulatory T cells with chronic activity and viral clearance in patients with hepatitis B. Int. Immunol. 19:133–140. 17182968 10.1093/intimm/dxl130

[16] Ward, S. M. , B. C. Fox , P. J. Brown , J. Worthington , S. B. Fox , R. W. Chapman , K. A. Fleming , A. H. Banham , and P. Klenerman . 2007 Quantification and localisation of FOXP3^+^ T lymphocytes and relation to hepatic inflammation during chronic HCV infection. J. Hepatol. 47:316–324. 17475362 10.1016/j.jhep.2007.03.023

[17] Nelson, D. R. , R. P. Gonzalez‐Peralta , K. Qian , Y. Xu , C. G. Marousis , G. L. Davis , and J. Y. Lau . 1997 Transforming growth factor‐b_1_ in chronic hepatitis C. J. Viral. Hepat. 4:29–35. 9031062 10.1046/j.1365-2893.1997.00124.x

[18] Mirsky, M. L. , C. A. Olmstead , Y. Da , and H. A. Lewin . 1996 The prevalence of proviral bovine leukemia virus in peripheral blood mononuclear cells at two subclinical stages of infection. J. Virol. 70:2178–2183. 8642640 10.1128/jvi.70.4.2178-2183.1996 PMC190056

[19] Schwartz, I ., and D. Lévy . 1994 Pathobiology of bovine leukemia virus. Vety Res 25:521–536. 7889034

[20] Frie, M. C. , and P. M. Coussens . 2015 Bovine leukemia virus: a major silent threat to proper immune responses in cattle. Vet. Immunol. Immunopathol. 163:103–114. 25554478 10.1016/j.vetimm.2014.11.014

[21] Gillet, N ., A. Florins , M. Boxus , C. Burteau , A. Nigro , F. Vandermeers , H. Balon , A. B. Bouzar , J. Defoiche , A. Burny , et al. 2007 Mechanisms of leukemogenesis induced by bovine leukemia virus: prospects for novel anti‐retroviral therapies in human. Retrovirology 4:18. 17362524 10.1186/1742-4690-4-18 PMC1839114

[22] Kabeya, H ., K. Ohashi , and M. Onuma . 2001 Host immune responses in the course of bovine leukemia virus infection. J. Vet. Med. Sci. 63:703–708. 11503896 10.1292/jvms.63.703

[23] Konnai, S ., T. Usui , K. Ohashi , and M. Onuma . 2003 The rapid quantitative analysis of bovine cytokine genes by real‐time RT‐PCR. Vet. Microbiol. 94:283–294. 12829382 10.1016/s0378-1135(03)00119-6

[24] Ikebuchi, R ., S. Konnai , T. Shirai , Y. Sunden , S. Murata , M. Onuma , and K. Ohashi . 2011 Increase of cells expressing PD‐L1 in bovine leukemia virus infection and enhancement of anti‐viral immune responses *in vitro* via PD‐L1 blockade. Vet. Res. 42:103. 21943148 10.1186/1297-9716-42-103 PMC3195098

[25] Suzuki, S ., S. Konnai , T. Okagawa , R. Ikebuchi , T. Shirai , Y. Sunden , C. N. Mingala , S. Murata , and K. Ohashi . 2013 Expression analysis of Foxp3 in T cells from bovine leukemia virus infected cattle. Microbiol. Immunol. 57:600–604. 23945026 10.1111/1348-0421.12073

[26] Suzuki, S ., S. Konnai , T. Okagawa , R. Ikebuchi , A. Nishimori , J. Kohara , C. N. Mingala , S. Murata , and K. Ohashi . 2015 Increased expression of the regulatory T cell‐associated marker CTLA‐4 in bovine leukemia virus infection. Vet. Immunol. Immunopathol. 163:115–124. 25618590 10.1016/j.vetimm.2014.10.006

[27] Panei, C. J. , S. N. Takeshima , T. Omori , T. Nunoya , W. C. Davis , H. Ishizaki , K. Matoba , and Y. Aida . 2013 Estimation of bovine leukemia virus (BLV) proviral load harbored by lymphocyte subpopulations in BLV‐infected cattle at the subclinical stage of enzootic bovine leucosis using BLV‐CoCoMo‐qPCR. BMC Vet. Res. 9:95. 23641811 10.1186/1746-6148-9-95 PMC3648496

[28] Ikebuchi, R ., S. Konnai , T. Okagawa , K. Yokoyama , C. Nakajima , Y. Suzuki , S. Murata , and K. Ohashi . 2013 Blockade of bovine PD‐1 increases T cell function and inhibits bovine leukemia virus expression in B cells *in vitro* . Vet. Res. 44:59. 23876077 10.1186/1297-9716-44-59 PMC3726328

[29] Storset, A. K. , S. Kulberg , I. Berg , P. Boysen , J. C. Hope , and E. Dissen . 2004 NKp46 defines a subset of bovine leukocytes with natural killer cell characteristics. Eur. J. Immunol. 34:669–676. 14991596 10.1002/eji.200324504

[30] Hoek, A ., V. P. Rutten , J. Kool , G. J. Arkesteijn , R. J. Bouwstra , I. Van Rhijn , and A. P. Koets . 2009 Subpopulations of bovine WC1(+) gammadelta T cells rather than CD4(+)CD25(high) Foxp3(+) T cells act as immune regulatory cells *ex vivo* . Vet. Res. 40:6. 18928784 10.1051/vetres:2008044 PMC2695017

[31] Snider T. G. 3rd , D. G. Luther , B. F. Jenny , P. G. Hoyt , J. K. Battles , W. H. Ennis , J. Balady , U. Blas‐Machado , T. X. Lemarchand , and M. A. Gonda . 1996 Encephalitis, lymphoid tissue depletion and secondary diseases associated with bovine immunodeficiency virus in a dairy herd. Comp. Immunol. Microbiol. Infect. Dis. 19:117–131. 8814974 10.1016/0147-9571(95)00032-1

[32] Gutiérrez, G ., S. M. Rodríguez , A. de Brogniez , N. Gillet , R. Golime , A. Burny , J. P. Jaworski , I. Alvarez , L. Vagnoni , K. Trono , and L. Willems . 2014 Vaccination against δ‐retroviruses: the bovine leukemia virus paradigm. Viruses 6:2416–2427. 24956179 10.3390/v6062416 PMC4074934

[33] Coussens, P. M. , S. Sipkovsky , B. Murphy , J. Roussey , and C. J. Colvin . 2012 Regulatory T cells in cattle and their potential role in bovine paratuberculosis. Comp. Immunol. Microbiol. Infect. Dis. 35:233–239. 22285689 10.1016/j.cimid.2012.01.004

[34] Guzman, E ., J. Hope , G. Taylor , A. L. Smith , C. Cubillos‐Zapata , and B. Charleston . 2014 Bovine γδ T cells are a major regulatory T cell subset. J. Immunol. 193:208–222. 24890724 10.4049/jimmunol.1303398 PMC4065783

[35] Pyeon, D ., K. L. O'Reilly , and G. A. Splitter . 1996 Increased interleukin‐10 mRNA expression in tumor‐bearing or persistently lymphocytotic animals infected with bovine leukemia virus. J. Virol. 70:5706–5710. 8764093 10.1128/jvi.70.8.5706-5710.1996 PMC190539

[36] Keefe, R. G. , D. A. Ferrick , and J. L. Stott . 1997 Cytokine transcription in lymph nodes of cattle in different stages of bovine leukemia virus infection. Vet. Immunol. Immunopathol. 59:271–283. 9477477 10.1016/s0165-2427(97)00083-4

[37] Pyeon, D ., F. J. Diaz , and G. A. Splitter . 2000 Prostaglandin E(2) increases bovine leukemia virus tax and pol mRNA levels via cyclooxygenase 2: regulation by interleukin‐2, interleukin‐10, and bovine leukemia virus. J. Virol. 74:5740–5745. 10823885 10.1128/jvi.74.12.5740-5745.2000 PMC112065

[38] Yakobson, B ., J. Brenner , H. Ungar‐Waron , and Z. Trainin . 2000 Cellular immune response cytokine expression during the initial stage of bovine leukemia virus (BLV) infection determines the disease progression to persistent lymphocytosis. Comp. Immunol. Microbiol. Infect. Dis. 23:197–208. 10855665 10.1016/s0147-9571(99)00074-0

[39] Trinchieri, G. 1989 Biology of natural killer cells. Adv. Immunol. 47:187–376. 2683611 10.1016/S0065-2776(08)60664-1 PMC7131425

[40] Mavilio, D ., J. Benjamin , M. Daucher , G. Lombardo , S. Kottilil , M. A. Planta , E. Marcenaro , C. Bottino , L. Moretta , A. Moretta , and A. S. Fauci . 2003 Natural killer cells in HIV‐1 infection: dichotomous effects of viremia on inhibitory and activating receptors and their functional correlates. Proc. Natl. Acad. Sci. U.S.A. 100:15011–15016. 14645713 10.1073/pnas.2336091100 PMC299884

[41] Lee, J. C. , K. M. Lee , D. W. Kim , and D. S. Heo . 2004 Elevated TGF‐β1 Secretion and Down‐Modulation of NKG2D Underlies Impaired NK Cytotoxicity in Cancer Patients. J. Immunol. 172:7335–7340. 15187109 10.4049/jimmunol.172.12.7335

[42] Giavedoni, L. D. , M. C. Velasquillo , L. M. Parodi , G. B. Hubbard , and V. L. Hodara . 2000 Cytokine expression, natural killer cell activation, and phenotypic changes in lymphoid cells from rhesus macaques during acute infection with pathogenic simian immunodeficiency virus. J. Virol. 74:1648–1657. 10644334 10.1128/jvi.74.4.1648-1657.2000 PMC111639

[43] Korbel, D. S. , K. C. Newman , C. R. Almeida , D. M. Davis , and E. M. Riley . 2005 Heterogeneous human NK cell responses to Plasmodium falciparum‐infected erythrocytes. J. Immunol. 175:7466–7473. 16301654 10.4049/jimmunol.175.11.7466

[44] Nilsson, J ., A. Boasso , P. A. Velilla , R. Zhang , M. Vaccari , G. Franchini , G. M. Shearer , J. Andersson , and C. Chougnet . 2006 HIV‐1‐driven regulatory T‐cell accumulation in lymphoid tissues is associated with disease progression in HIV/AIDS. Blood 108:3808–3817. 16902147 10.1182/blood-2006-05-021576 PMC1895475

[45] Harhaj, E. W. , N. S. Harhaj , C. Grant , K. Mostoller , T. Alefantis , S. C. Sun , and B. Wigdahl . 2005 Human T cell leukemia virus type I Tax activates CD40 gene expression via the NF‐kappa B pathway. Virology 333:145–158. 15708600 10.1016/j.virol.2004.12.008

[46] Twizere, J. C. , V. Kruys , L. Lefèbvre , A. Vanderplasschen , D. Collete , C. Debacq , W. S. Lai , J. C. Jauniaux , L. R. Bernstein , O. J. Semmes , et al. 2003 Interaction of retroviral Tax oncoproteins with tristetraprolin and regulation of tumor necrosis factor‐alpha expression. J. Natl. Cancer Inst. 95:1846–1859. 14679154 10.1093/jnci/djg118

[47] Francisco, L. M. , V. H. Salinas , K. E. Brown , V. K. Vanguri , G. J. Freeman , V. K. Kuchroo , and A. H. Sharpe . 2009 PD‐L1 regulates the development, maintenance, and function of induced regulatory T cells. J. Exp. Med. 206:3015–3029. 20008522 10.1084/jem.20090847 PMC2806460

